# Tubulin binding potentially clears up Bortezomib and Carfilzomib differential neurotoxic effect

**DOI:** 10.1038/s41598-021-89856-3

**Published:** 2021-05-18

**Authors:** A. Malacrida, S. Semperboni, A. Di Domizio, A. Palmioli, L. Broggi, C. Airoldi, C. Meregalli, G. Cavaletti, G. Nicolini

**Affiliations:** 1 School of Medicine and Surgery, Experimental Neurology Unit, University of Milano - Bicocca, Via Cadore 48, 20900 Monza, MB Italy; 2 Milan Center for Neuroscience, University of Milano - Bicocca, Piazza dell’Ateneo Nuovo 1, 20126 Milan, MI Italy; 3 Department of Pharmacological and Biomolecular Sciences, University of Milano, Via Balzaretti 9, 20133 Milan, Italy; 4 SPILLOproject, Via Stradivari 17, Paderno Dugnano, 20037 Milano, Italy; 5 Department of Biotechnology and Biosciences, BioOrgNMR Lab, University of Milano - Bicocca, P.zza della Scienza 2, 20126 Milan, Italy

**Keywords:** Cancer therapy, Cytoskeleton, Mechanisms of disease

## Abstract

Proteasome inhibitors (PIs) represent the gold standard in the treatment of multiple myeloma. Among PIs, Bortezomib (BTZ) is frequently used as first line therapy, but peripheral neuropathy (PN), occurring approximately in 50% of patients, impairs their life, representing a dose-limiting toxicity. Carfilzomib (CFZ), a second-generation PI, induces a significantly less severe PN. We investigated possible BTZ and CFZ off-targets able to explain their different neurotoxicity profiles. In order to identify the possible PIs off-targets we used the SPILLO-PBSS software that performs a structure-based in silico screening on a proteome-wide scale. Among the top-ranked off-targets of BTZ identified by SPILLO-PBSS we focused on tubulin which, by contrast, did not turn out to be an off-target of CFZ. We tested the hypothesis that the direct interaction between BTZ and microtubules would inhibit the tubulin alfa GTPase activity, thus reducing the microtubule catastrophe and consequently furthering the microtubules polymerization. This hypothesis was validated in a cell-free model, since BTZ (but not CFZ) reduces the concentration of the free phosphate released during GTP hydrolysis. Moreover, NMR binding studies clearly demonstrated that BTZ, unlike CFZ, is able to interact with both tubulin dimers and polymerized form. Our data suggest that different BTZ and CFZ neurotoxicity profiles are independent from their proteasome inhibition, as demonstrated in adult mice dorsal root ganglia primary sensory neurons, and, first, we demonstrate, in a cell free model, that BTZ is able to directly bind and perturb microtubules.

## Introduction

**Figure 1:**
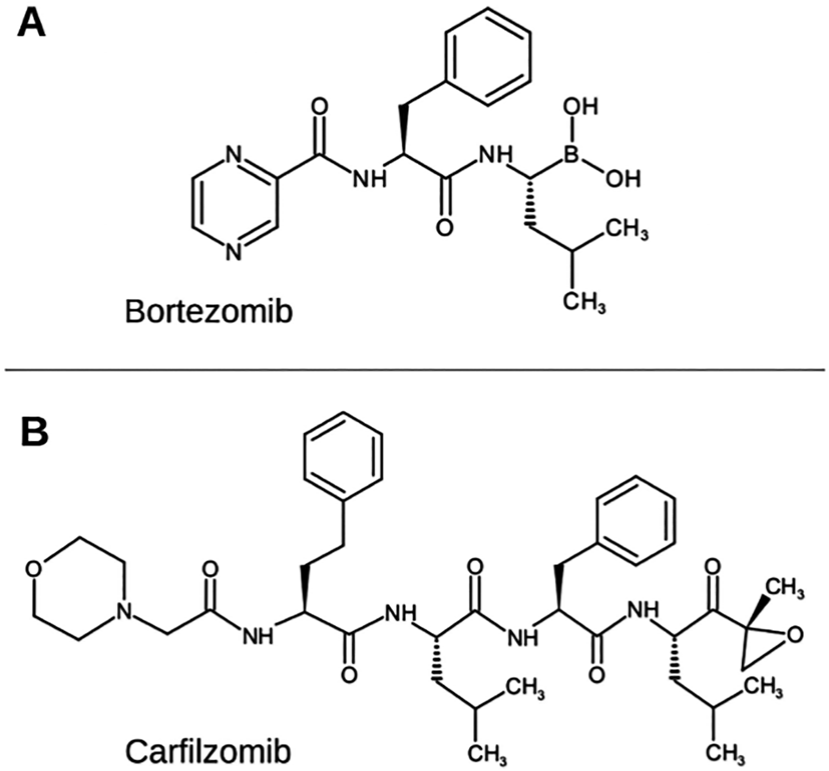
Bortezomib and Carfilzomib. Chemical structures of Bortezomib (**A**) and Carfilzomib (**B**) are reported (structures drawn using ACD/ChemSketch^14^).

The different neurotoxicity profiles of the proteasome inhibitors (PIs) Bortezomib (BTZ) and Carfilzomib (CFZ) suggest that BTZ more severe neurotoxic effect might be due, at least partially, to a proteasome-independent pathway. The contribution of the cytoskeletal damage, chaperone system and protein oxidation pathways, as well as protein K48-ubiquitinaton, serine proteases inhibition and heat shock protein upregulation was associated with higher rates of BTZ-induced neurotoxicity than CFZ^1–3^. Both BTZ (a dipeptide boronic acid derivative) and CFZ (a tetrapeptide epoxyketone) (Fig. 1), cornerstones in the treatment of multiple myeloma (MM), are able to inhibit the chymotrypsin-like subunit perturbing myeloma cell signalling and inducing cell apoptosis. However, the use of BTZ as a first line drug in MM, especially in prolonged therapy, is limited by the development of neuropathy associated with severe neuropathic pain. On the contrary, the use of CFZ in patients pretreated with at least one BTZ containing schedule does not increase significantly PN (especially high grades) and does not cause treatment interruption^4^.

Chemotherapy-induced peripheral neuropathy (CIPN), a common side effect of different classes of antineoplastic agents, has an etiology still not fully understood. In fact, for many chemotherapy drugs, their antineoplastic effect cannot be directly related to their neurotoxic effect (e.g. platinum derivatives). Considering that CIPN is often a limiting side effect on the chemotherapeutic treatment, many studies have investigated possible antineoplastic drugs (including proteasome inhibitors) off-targets^5,6^. In animal models BTZ is able to induce tubulin hyperpolymerization^7^, and under this perspective it is fundamental to clarify its effect(s) on neuron cytoskeleton architecture. In fact, neurons, with their polarized morphology, are strictly dependent for their functioning and survival on the right assembling, structure and dynamic remodelling of microtubule (MT), microfilaments and intermediate filaments. All three components of cytoskeleton are able to influence all neurons functions^6,8^.

In particular MT dynamics are carefully regulated by post-translational modifications (acetylation, phosphorylation, polyglycylation, detyrosination, delta2 tubulin and polyglutamylation), interaction with microtubule-associated proteins (MAPs) and polymer and dimer ratio are in particular perturbed by antineoplastic drugs classified as microtubule targeting agents (MTAs)^6^. Moreover, strong evidence of the pathogenic role of delta2 accumulation was recently demonstrated in sensory neurons in in vivo and in vitro studies of BTZ-induced peripheral neuropathy. These changes were found to be related to axonal MT stabilization, mitochondrial energetics impairment, and they are suggested as sufficient and necessary conditions to cause axonopathy with selective alterations of mitochondria motility^9^. In this study, we demonstrated that BTZ and CFZ doses, resulting cytotoxic for multiple myeloma cells and able to inhibit proteasome in the same manner in adult mouse DRG sensory neurons, show in this cellular model the same different neurotoxicity profiles observed in clinical practice. Then, a 3D proteome-wide scale in silico screening of a database including protein structures of *Homo sapiens*, *Mus musculus* and *Rattus norvegicus* retrieved from the RCSB Protein Data Bank^10^ was performed using SPILLO-PBSS^11–13^ and tubulin was identified as a direct off-target of BTZ but not of CFZ. Finally, we validated the results in a cell free model and by investigating BTZ and CFZ binding to tubulin through NMR spectroscopy.

## Results

### U266.B1 cell viability

In order to find BTZ and CFZ IC_50_, U266.B1 cells were treated for 48 h with increasing drugs’ concentrations (1–10 nM BTZ; and 0.5–10 nM CFZ) and MTT and Trypan blue vital count assay were performed. Untreated cells were used as control.

**Figure 2:**
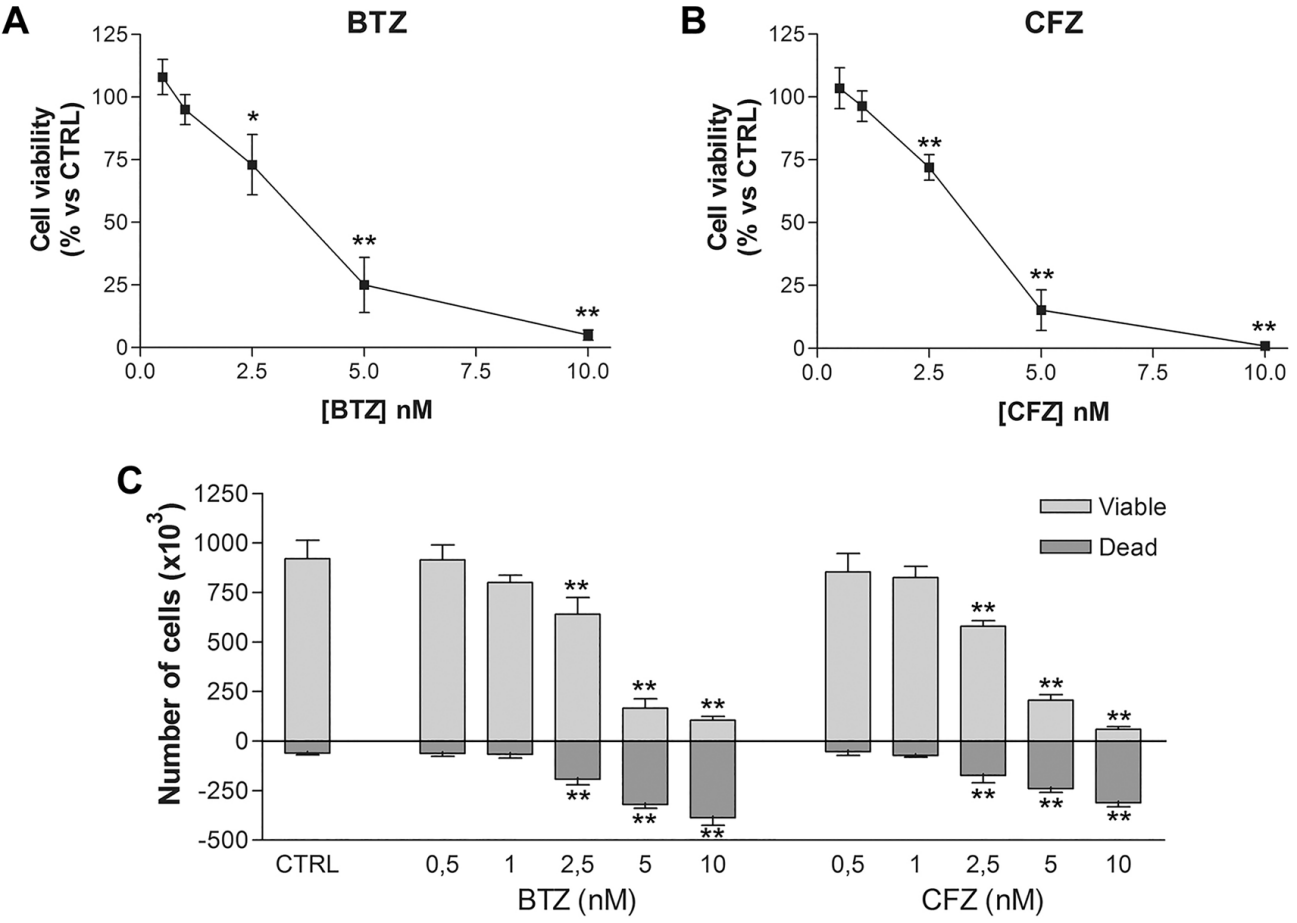
Cell viability of U266.B1 cells after BTZ and CFZ treatment. (**A**) MTT assay of U266.B1 cells treated for 48 h with increasing concentrations of BTZ (0.5–10 nM) or (**B**) CFZ (0.5–10 nM). (**C**) Viable and dead U266.B1 cells counted by Trypan blue vital count after 48 h of treatment with increasing concentrations of BTZ and CFZ (0.5–10 nM). All graphs are represented as mean ± SD of three independent experiments and are compared to untreated CTRL. *p < 0.05, **p < 0.01 versus CTRL.

As shown in Fig. 2A,B, both BTZ and CFZ impaired U266.B1 cells viability, at 48 h, in a dose dependent manner. IC_50_ (derived from the graphs) is equal to 2.8 nM and 3.2 nM for BTZ and CFZ, respectively.

Trypan blue vital count (Fig. 2C) confirmed the results obtained with MTT assay, but also highlighted that the total cells reduction observed with BTZ and CFZ treatment is due to cellular death (as shown by the number of dead cells counted) and not to a reduction of proliferation.

### Neurons viability

**Figure 3:**
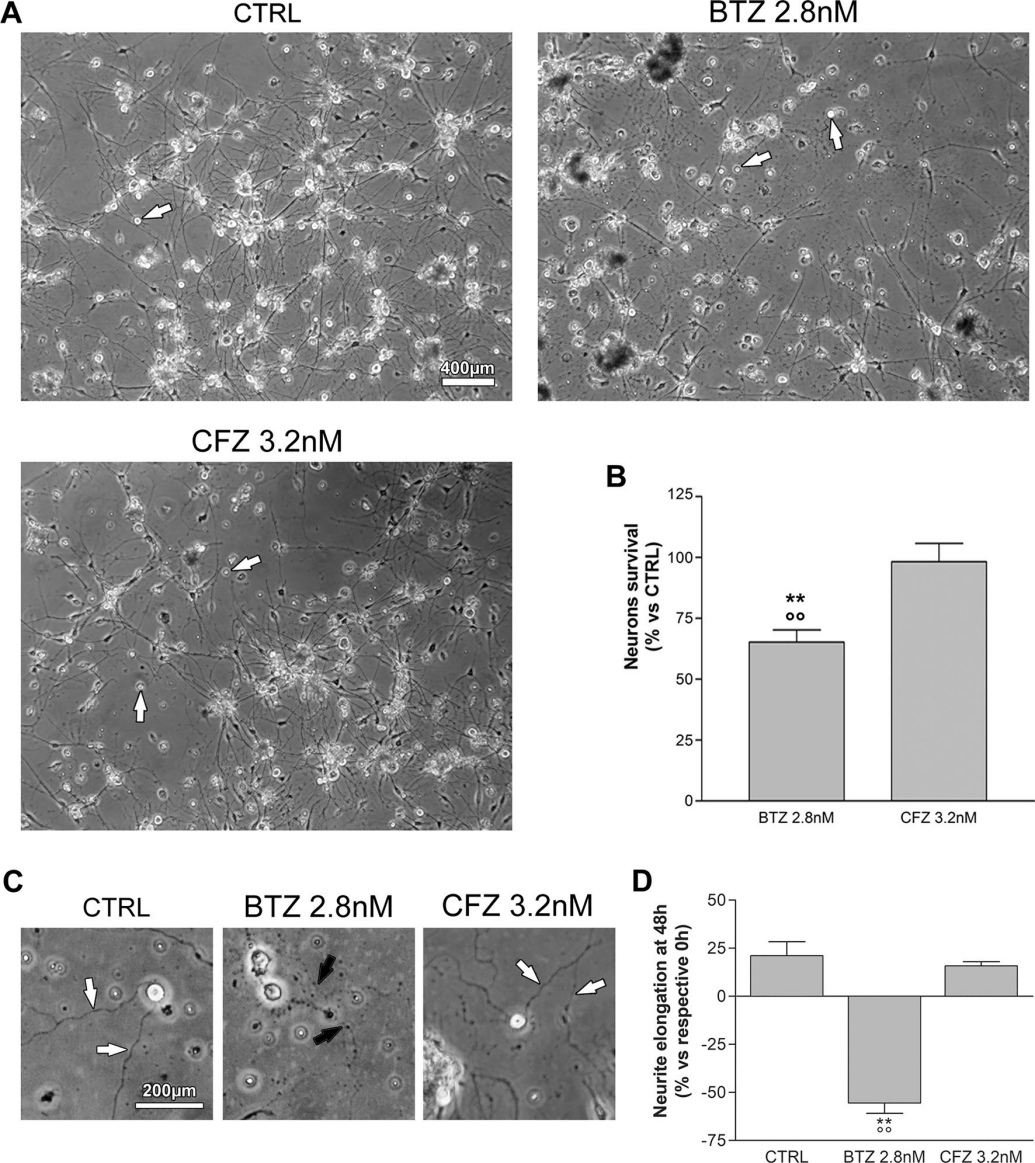
Neuron cultures viability after treatment with BTZ and CFZ. (**A**) Representative images of neuron cultures treated for 48 h with 2.8 nM BTZ or 3.2 nM CFZ or untreated (CTRL). Bar = 400 μm. White arrows indicate viable neurons. (**B**) Graph represents the percentage of viable neurons after treatment with BTZ 2.8 nM and CFZ 3.2 nM and are compared to untreated CTRL arbitrarily set to 100%. (**C**) Representative images of normal neurites (CTRL and CFZ 3.2 nM, white arrows) and degenerated neurites (BTZ 2.8 nM, black arrows). Bar = 200 μm. (**D**) The graph represents the elongation of neurites untreated (CTRL), treated with BTZ 2.8 nM or CFZ 3.2 nM, after 48 h. Percentages are calculated on the respective lengths at 0 h **p < 0.01 versus CTRL; °°*p* < 0.01 versus CFZ 3.2 nM.

In order to study the neurotoxicity of BTZ and CFZ MM IC_50_, adult mice DRG sensory neurons primary cultures were treated for 48 h with BTZ (2.8 nM) and CFZ (3.2 nM) (Fig. 3A) and vital counts were evaluated (Fig. 3B). Untreated neurons were used as control. The results showed that BTZ induced a statistically significant (*p* < 0.01) increase in neuron mortality compared to untreated control, but also compared to CFZ treated neurons. Moreover, BTZ induced an evident reduction of neurites length (Fig. 3C, D). Otherwise, the viability of CFZ-treated neurons is comparable with the one of untreated control and their neurites were still well visible and branched.

### U266.B1 cells and sensory neurons proteasome study

In order to assess if BTZ and CFZ toxicity on U266 .B1 cells and sensory neurons was related to BTZ and CFZ proteasome inhibitory effect both cell models were treated with BTZ (2.8 nM) and CFZ (3.2 nM) for 48 h and proteasome activity was evaluated on total protein extracts through fluorometric assay.

**Figure 4:**
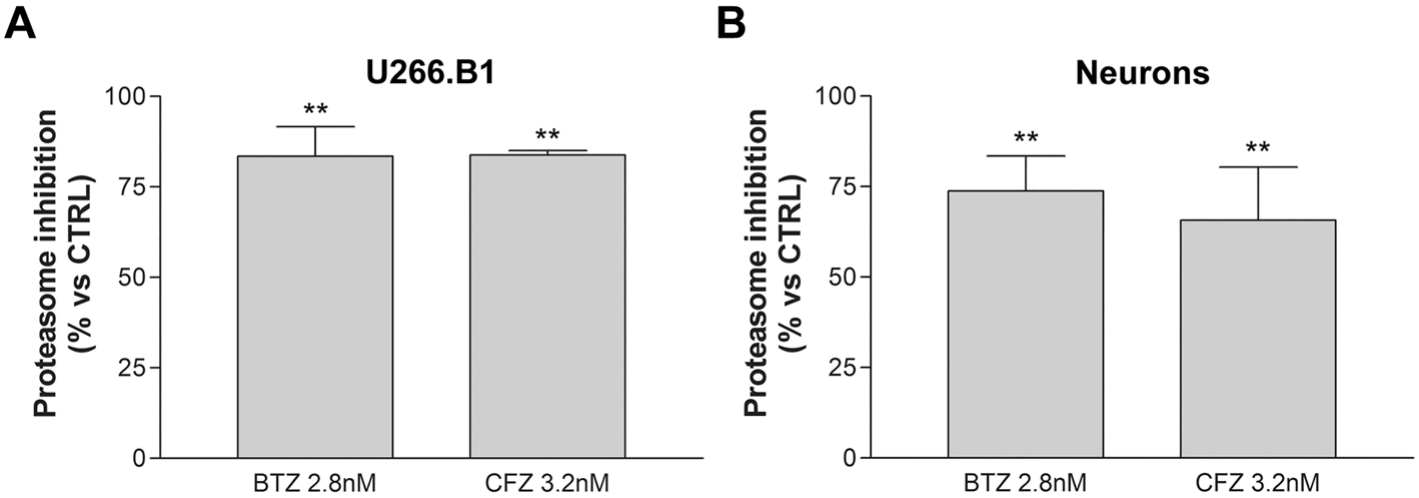
BTZ and CFZ induced proteasome inhibition in U266.B1 cells and sensory neurons. Proteasome inhibition of U266.B1 cells (**A**) and neurons (**B**) after treatment for 48 h with 2.8 nM BTZ or 3.2 nM CFZ. Graphs are represented as mean percentage ± SD compared to an untreated CTRL (arbitrarily set to 0%). **p < 0.01 versus CTRL

U266 .B1 cells treated with BTZ or CFZ show a statistically significant (*p* < 0.01) proteasome inhibition compared to untreated control. The proteasome inhibition induced by the two compounds was perfectly comparable (83.4% and 83.8% respectively) (Fig. 4A). As an unexpected result, both BTZ and CFZ induced a statistically significant (*p* < 0.01) proteasome inhibition in sensory neurons (73.8% and 65.7% respectively) compared to untreated control. In particular, no statistically significant differences were noticed between the two compounds in terms of proteasome inhibition effect (Fig. 4B).

### In silico protein database screening and ranking

**Figure 5:**
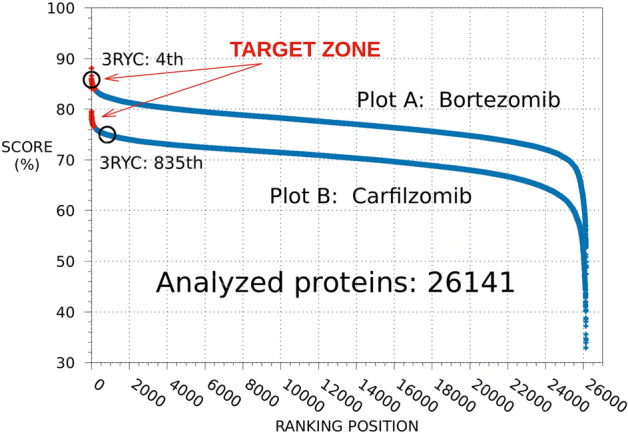
SPILLO-PBSS screenings for BTZ and CFZ. Plots resulting from the in silico screenings carried out for BTZ (Plot A) and CFZ (Plot B) on the available structural proteomes (26,141 protein 3D-structures retrieved from the RCSB PDB in August 2019, excluding 100% sequence identity redundancies) of *Homo sapiens*, *Mus musculus*, and *Rattus norvegicus*. Proteins are ranked in decreasing order according to their score. Tubulin (PDB code: 3RYC) was identified as a potential off-target for BTZ (4th position), but not for CFZ (835th position), which could account for the different neurotoxic profile of the two drugs (plots drawn using gnuplot^15^).

To identify potential off-target interactions of BTZ and CFZ able to account for their different neurotoxic profiles, we used the SPILLO-PBSS software to screen and rank a large protein database including the available structural proteomes (26,141 protein 3D-structures, August 2019) of three different organisms, namely *Homo sapiens*, *Mus musculus*, and *Rattus norvegicus*. It is worth noting that SPILLO-PBSS is not a molecular docking program and it is able to identify possible target (and off-target) proteins of any small molecule through a direct identification of their potential binding sites (PBSs), which are identified even when they are not in a conformation suitable for the binding (e.g., occupied by a ligand or completely closed). Indeed, SPILLO-PBSS performs an unbiased search of both the surface and the inner regions of the protein and compares the 3D structure of a binding site used as reference (the reference binding site, RBS) with that of any PBS present in the target protein on a similarity basis. Possible conformational rearrangements that may occur to the binding site upon binding of the small molecule are also taken into account^11–13^.This analysis led to the two plots reported in Fig. 5, whose points correspond to proteins ranked in decreasing order according to their scores (ranging from 0 to 100%), representing the highest similarity to the corresponding RBS, obtained from each analyzed protein 3D-structure. In particular, the non-linearity of the curves highlights the presence of a minority of proteins with scores clearly higher than all others, corresponding to the potential off-targets of the drugs. Interestingly, the average height of the CFZ curve is lower than that of BTZ, which could be expected considering that CFZ (a tetrapeptide epoxyketone) is larger than BTZ (a dipeptide boronic acid) and that the higher the number of amino acids in the RBS (see Supplementary Table S1), the lower the probability of identifying PBS similar to the reference in the analyzed proteins.

### Tubulin as potential off-target of bortezomib

Among the top-ranked potential off-targets of BTZ identified by SPILLO-PBSS, we focused our attention on tubulin (PDB code: 3RYC, 4th position), since neurotoxicity was already correlated with the tubulin hyperpolymerization, as previously published^7^. For this reason, although other proteins have also been differentially selected by the software, between BTZ and CFZ, we decided to start our experimental validations from tubulin, while an in-depth analysis and validation for other top-ranked off-target proteins will be the matter of future studies. Similarly to microtubule-targeting agents (MTA), PN could be associated with a perturbation of microtubule dynamics^16^. Although 3RYC includes the crystal structure of *Ovis aries* αβ-tubulin in the GDP and GTP nucleotide states in a complex with the *Rattus norvegicus* stathmin-like domain (SLD)^17^, BTZ interacts only with αβ-tubulin, without any involvement of SLD. Conversely, tubulin was not identified (835^th^ position) as an off-target for CFZ. This result allowed us to hypothesize a possible interference of BTZ with the delicate equilibria that regulate microtubule dynamics that may account for the differences in neurotoxic profile between BTZ and CFZ.

### Cross-organism transferability analysis

Since the aim of this work was to provide a possible interpretation of the different neurotoxic profile of BTZ and CFZ on humans, a cross-organism transferability analysis was carried out aimed at assessing the transferability of the result from *Ovis aries* to *Homo sapiens.* In particular, a positive assessment was provided by the overall sequence comparison (calculations performed by BLAST^18^) between the corresponding tubulin chains and isoforms of the two organisms, which resulted in a high (greater than or equal to 88.3%) percentage sequence identity for every comparison performed (see Supplementary Table S2).

The same kind of analysis was also performed for the tubulin chains of *Mus musculus*, from which derive the sensory neurons then used to study the effect of BTZ and CFZ on tubulin expression and polymerization, and for the tubulin chains of *Sus scrofa*, which were then used to study in vitro how BTZ and CFZ affect the dynamics of microtubules and to assess the direct interaction of BTZ and CFZ with tubulin by NMR (see next paragraphs). The high percentage sequence identities (greater than or equal to 90.0%) with the tubulin chains of *Ovis aries* allowed us to positively assess the reliability of the designed experiments to validate the SPILLO-PBSS-predicted off-target (see Supplementary Table S2).

### Off-target validation

#### Sensory neurons tubulin analysis

In order to study the possible effect of BTZ and CFZ on tubulin expression and polymerization in sensory neurons, cells were treated with drugs IC_50_ for 48 h and tubulin immunoblotting were performed on fractionated protein extracts.

**Figure 6:**
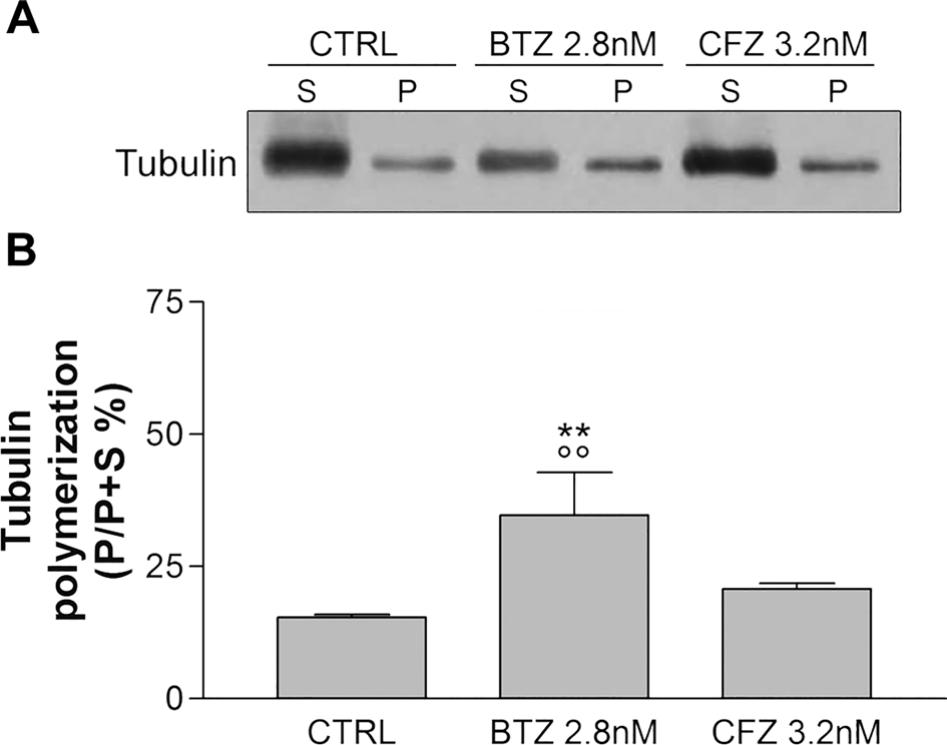
Tubulin polymerization in DRG neurons. Representative images (**A**) and quantification graphs (**B**) of anti-tubulin immunoblotting in neurons, not treated (CTRL) or treated for 48 h with 2.8 nM BTZ and 3.2 nM CFZ. Graphs represent the percentage of polymerized tubulin (present in the pellet fraction P) compared to total tubulin (free tubulin, present in the substrate fraction S, added to polymerized tubulin). **p < 0.01 versus CTRL; °°*p* < 0.01 versus CFZ 3.2 nM.

In untreated neurons, the amount of polymerized tubulin corresponds to 15.4%. 3.2 nM CFZ treatment slightly increases the percentage of polymerized tubulin in neurons, however not significantly compared to controls (20.8%). On the contrary, the treatment with 2.8 nM BTZ doubles the polymerization percentage to 34.7% in neurons (difference statistically significant versus untreated controls and CFZ treated, Fig. 6). The same increase is observed in U266.B1 myeloma cells following treatment with BTZ, but not with CFZ (Supplementary Figure S1).

#### In vitro tubulin polymerization and phosphate quantification

The dynamics of microtubules is linked to a GTP/GDP cycle and an important role in its regulation is played by GTPase catalytic site of β tubulin, responsible for the hydrolysis of GTP to GDP^19^. To evaluate whether this catalytic activity can be affected by the interaction between BTZ and tubulin identified by SPILLO-PBSS software, an immunoblotting against GTP-tubulin was performed.

**Figure 7:**
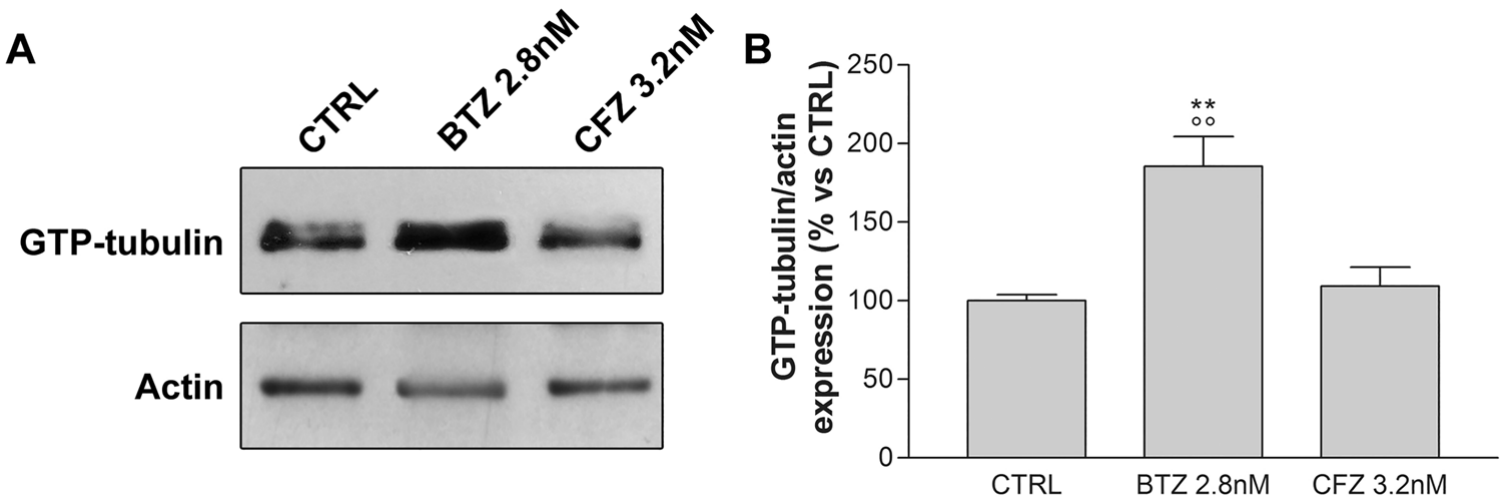
GTP-tubulin in sensory neurons. Representative image (**A**) and quantification graph (**B**) of GTP-tubulin immunoblotting in neurons not treated (CTRL) or treated for 48 h with 2.8 nM BTZ and 3.2 nM CFZ. Data are normalized respect to actin value and expressed as percentage respect control. **p < 0.01 versus CTRL; °°p < 0.01 versus CFZ 3.2 nM.

As shown in Fig. 7A, B, 2.8 nM BTZ significantly increases the percentage of GTP-tubulin compared to untreated control, while 3.2 nM CFZ does not induce any alteration.

To confirm this result and to demonstrate a direct interaction between BTZ and tubulin, an in vitro cell-free polymerization assay was performed. BTZ and CFZ concentrations were increased to reach at least a ratio, calculated on the number of molecules, of 1 to 20 between drugs and tubulin.

**Figure 8:**
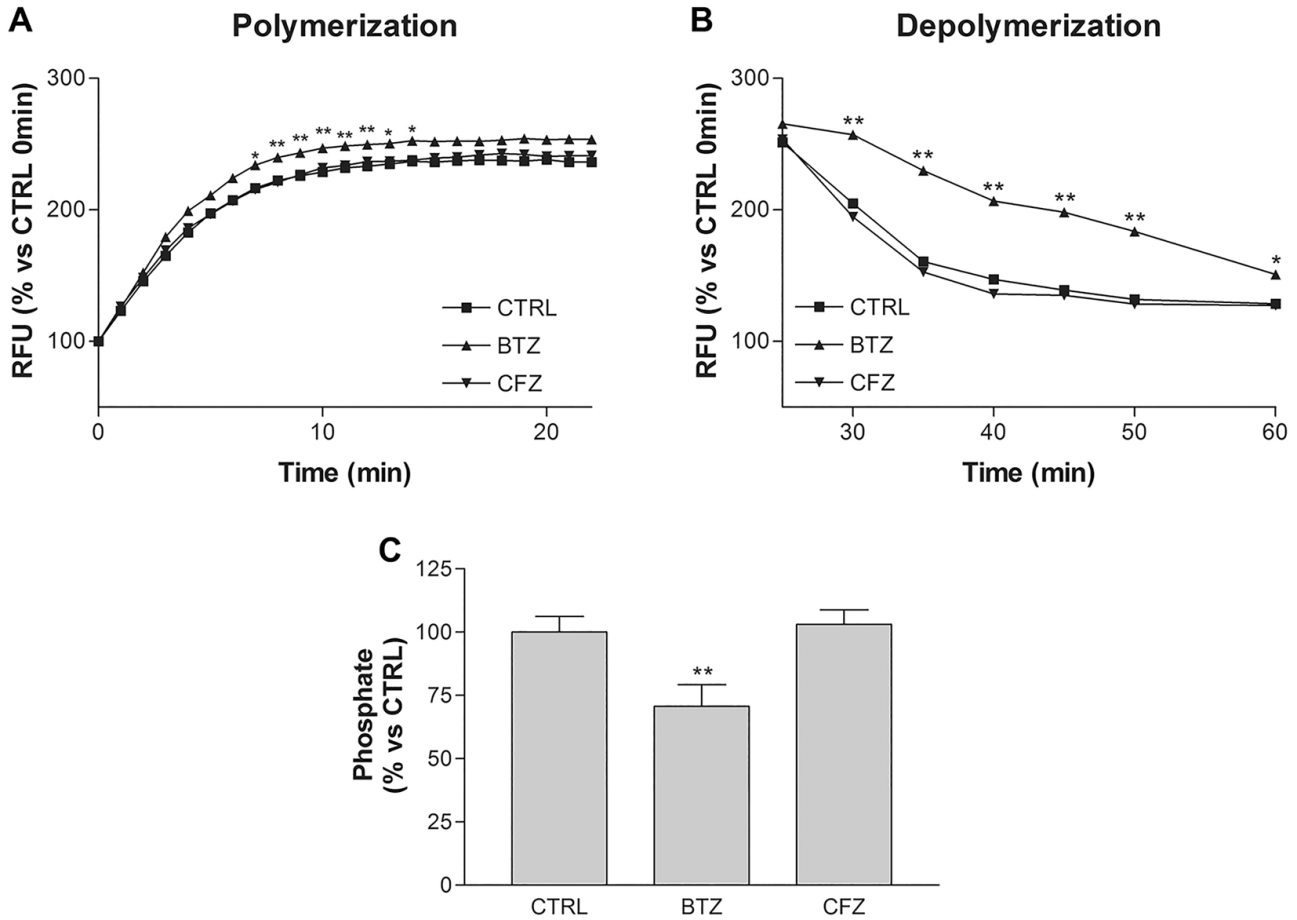
Tubulin polymerization and depolymerization in vitro with DMSO buffer. (**A**) In vitro cell-free tubulin polymerization in presence of 1 µM BTZ or 1 µM CFZ. (**B**) In vitro cell-free tubulin depolymerization in presence of 1 µM BTZ or 1 µM CFZ. (**C**) Phosphate quantification after tubulin polymerization assay. Graphs are represented as mean percentage ± SD compared to untreated CTRL (arbitrarily set to 100%). *p < 0.05; **p < 0.01 versus CTRL and versus CFZ.

Based on literature data, 8% DMSO is the best concentration to induce an in vitro cell-free physiologic-like tubulin polymerization^20^. Unlike glycerol, DMSO induces a faster polymerization of tubulin: the polymerization lag is 0 min, the reaction reaches the plateau after 10 min and the polymerization rate is 19.57 RFU%/min. As shown in Fig. 8A, CFZ has no effect on tubulin polymerization and samples treated with this drug have polymerization rate comparable to untreated control. BTZ instead has a slight effect on tubulin polymerization: it increases polymerization rate to 23.62 RFU%/min (Fig. 8A, Table 1).

**Table 1:** Tubulin polymerization and depolymerization rate and lag.

	Polymerization rate RFU% / min	Polymerization lag min	Depolymerization rate RFU% / min	Depolymerization lag min
**DMSO buffer**				
CTRL	19.57 ± 0.29	0	9.05 ± 0.65	2.11 ± 0.25
BTZ	23.62 ± 0.69 **	0	3.50 ± 0.12 **	9.45 ± 0.76 **
CFZ	19.52 ± 0.88	0	10.07 ± 0.85	2.29 ± 0.87

**p < 0.01 versus CTRL and CFZ.

Depolymerization of tubulin occurs when the plate is placed at 4 °C. In untreated CTRL, tubulin starts to depolymerize after 2.11 min with a depolymerization rate of 9.05 RFU%/min. CFZ does not influence tubulin depolymerization and it is comparable to untreated control. BTZ, instead, significantly slows down tubulin depolymerization: depolymerization lag is 9.45 min and the rate is 3.50 RFU%/min (Fig. 8B, Table 1).

Since the only phosphate source within the reaction solution is given by the hydrolysis of GTP to GDP, it can be considered a good indicator of β tubulin catalytic activity. An aliquot was taken from the solution of the previous polymerization reaction to evaluate the amount of free phosphate. Measured levels in CFZ condition are comparable to untreated controls. On the contrary, BTZ induces a significant reduction of free phosphate (Fig. 8C), thus proving that it slows down tubulin depolymerization by acting as a tubulin GTPase inhibitor. In turn, Paclitaxel induces a significant increase in polymerization and a greater stability of the tubulin polymerized form at low temperatures, compared to both untreated control, BTZ and CFZ. On the other hand Paclitaxel does not alter the production of free phosphate (Supplementary Figure S2).

#### NMR-based evaluation/assessment of BTZ and CFZ interaction with tubulin

BTZ and CFZ binding to tubulin was evaluated through STD (Saturation Transfer Difference) NMR experiments^21^.

**Figure 9:**
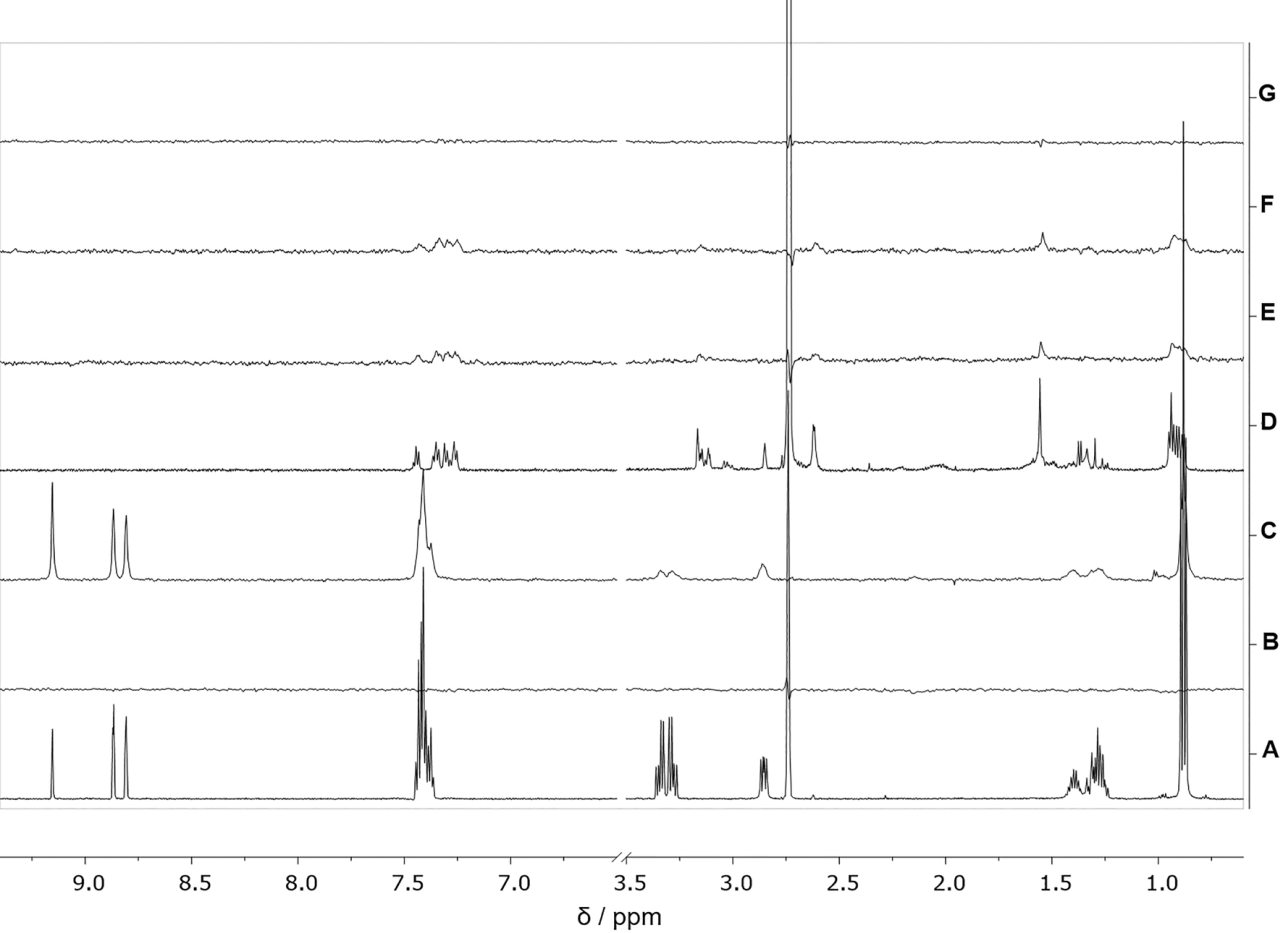
NMR spectra. (**A**) ^1^H NMR spectrum of 500 μM BTZ dissolved in 20 mM PB, MgCl_2_, pH 7.4, d_6_-DMSO 8% v/v, 37 °C; (**B**) STD NMR spectrum of 500 μM BTZ dissolved in 20 mM PB, MgCl_2_, pH 7.4, d_6_-DMSO 8% v/v, 37 °C; (**C**) STD NMR spectrum of 500 μM BTZ and tubulin α/β dimers 10 μM in polymeric form dissolved in 20 mM PB, MgCl_2_, GTP 100 μM, pH 7.4, d_6_-DMSO 8% v/v, 37 °C; (**D**) ^1^H NMR spectrum of 500 μM CFZ dissolved in 20 mM PB, MgCl2, pH 7.4, d_6_-DMSO 8% v/v, 37 °C; (**E**) STD NMR spectrum of 500 μM CFZ dissolved in 20 mM PB, MgCl_2_, pH 7.4, d_6_-DMSO 8% v/v, 37 °C; (**F**) STD NMR spectrum of 500 μM CFZ and tubulin α/β dimers 10 μM in polymeric form dissolved in 20 mM PB, MgCl_2_, GTP 100 μM, pH 7.4, d_6_-DMSO 8% v/v, 37 °C; (**G**) STDD NMR spectrum obtained by subtraction of spectrum E from spectrum F. All spectra were acquired at 600 MHz after at least 30 min sample incubation at 37 °C.

Firstly, we investigated drugs’ interaction with tubulin polymeric form. Briefly, to tubulin samples (10 μM or 20 μM, nominal α/β dimer concentration dissolved in 20 mM PB buffer, pH 7.4, containing 1 mM MgCl_2_ and 100 μM GTP), BTZ or CFZ were added at a final concentration of 500 μM, after their dissolution in d_6_-DMSO. In fact, while BTZ shows a good water solubility, CFZ solubility is very poor, requiring the use of d_6_-DMSO as co-solvent to be dissolved at a concentration sufficient for NMR spectra acquisition. Moreover, DMSO is reported to induce tubulin polymerization, increasing its rate and stabilizing tubulin polymeric form^22,23^. Thus, the addition of DMSO was exploited for both CFZ solubilization and tubulin polymerization. According to previous data^20^, the optimal DMSO concentration corresponds to 8% v/v in water (or aqueous buffer) that was thus used for NMR sample preparation. Moreover, to assure the polymeric state of tubulin during NMR spectra acquisition, each sample was incubated at 37 °C for at least 30 min. Each mixture was analyzed irradiating the sample at − 1.0 ppm (Fig. 9) or − 2.0 ppm (data not shown) to achieve the selective saturation of some aliphatic resonances of tubulin. In general, the presence of NMR signals of the test molecule in the STD spectra is a non-ambiguous demonstration of the existence of interaction. Conversely, the absence of NMR resonances in the STD spectra indicates that the molecule is not a tubulin ligand. The STD NMR spectra recorded for BTZ or CFZ and in the presence of tubulin polymeric form, at 10 μM (Fig. 9, spectra C and F, respectively) or 20 μM (data not shown) showed test compounds’ resonances. Notably, the blank STD spectrum, recorded for BTZ, by acquiring the same experiment in the absence of tubulin, did not contain resonances, confirming that the STD signals appearing in spectrum C (Fig. 8) were due to BTZ interaction with tubulin polymeric form. Instead, the blank STD spectrum recorder for CFZ (Fig. 9, spectrum E) was identical to that acquired in the presence of tubulin. This observation was confirmed by the STDD (Saturation Transfer Double Difference) spectrum, obtained subtracting spectrum XE from spectrum XF (Fig. 9G), as it did not show CFZ resonances. This means that the CFZ STD signals present in spectrum F (Fig. 9) are artifacts, probably due to CFZ aggregation, and are not consequence of CFZ binding to tubulin. All together, these findings clearly show that, while BTZ recognizes and bind tubulin assemblies, CFZ does not.

Moreover, we also investigated BTZ interaction with tubulin α/β dimers. The NMR sample was prepared as previously described, but without DMSO addition, and it was put at 10 °C. BTZ was dissolved in water and added to the sample immediately before NMR experiments’ acquisition, which was performed while keeping the sample at 10 °C. Under these experimental conditions, tubulin polymerization does not occur. The corresponding STD NMR spectrum is depicted in Supplementary Figure S3B. The presence of BTZ resonances indicated unambiguously that the drug interacts also with the dimeric form of tubulin (Supplementary Figure S3).

## Discussion

CIPN is a common dose-limiting side effect of several antineoplastic drugs, belonging to different classes, that negatively affects patients’ morbidity and quality of life. Considering the increase in percentage of cancer survival due to the development of more effective treatment, CIPN is turning as a medical topic crucial for the management of cancer patients. Unfortunately, currently very little is known about the pathogenesis of CIPN and, among all the preventive/therapeutic strategies tested, none proved to be resolutive. In the present work, we have investigated BTZ and CFZ, two proteasome inhibitors that are characterized by different neurotoxic profiles in clinical practice. The hypothesis that the neurotoxicity of chemotherapy is due to the interaction of drugs with off-target molecules is becoming more and more consistent. In particular several studies have investigated different proteasome inhibitors’ off-targets which could be responsible for neurotoxic effects^1,24^. Arastu-Kapur et al. (2011) studied off-target activity of equivalent levels of proteasome inhibition induced by BTZ and CFZ in retinoic-acid differentiated SH-SY5Y cells, in which only BTZ induced neurite degeneration. In addition, inhibition of cathepsin G in splenocytes of rats and peripheral blood mononuclear cells collected from BTZ-treated patients were also reported. Finally, BTZ, but not CFZ, is reported as a potent inhibitor of a prosurvival protease HtrA2/Omi in SH-SY5Y cells, suggesting mitochondria toxicity as a possible indirect effect of BTZ. These results suggested for the first time several proteasome-independent mechanisms involved in BTZ-neurotoxicity^1^. More recently, a proteomics approach to explain the different neurotoxic effects induced by BTZ and CFZ was performed in mouse neural stem cells, evidencing higher impairment of cytoskeletal proteins, related stress response systems and protein oxidation after BTZ-treatment than CFZ^2^. In addition, the same working group extended previous results on identification of affected proteins following BTZ and CFZ treatment using LC–MS based proteomics studies in human neuronal cells. The similar targets were investigated, confirming that after 24 h of chemotherapy treatments the levels in heat shock response (HSP70, HSP32) was higher extended in BTZ than CFZ, and a different proteotoxic response was observed after BTZ and CFZ treatment. Moreover, only BTZ was able to induce the loss of mitochondrial membrane potential induced in neuronal cells, although BTZ and CFZ cause a comparable mitotoxicity event, demonstrating that the different toxic profile between BTZ and CFZ was not due to mitochondria-related pathways^3^.

In our studies, we compared the neurotoxicity of BTZ and CFZ at *c*oncentrations resulting in cytotoxicity for multiple myeloma cells and able to inhibit proteasome in the same manner in adult mouse DRG sensory neurons in vitro models. At these concentrations, and in this cellular model, the two drugs showed the same different neurotoxicity profiles observed in clinical practice.

In order to identify the molecular off-target allowing BTZ to affect sensory neurons viability, we used SPILLO-PBSS, a powerful software for identifying targets and off-targets of any small molecule on proteome-wide scale. SPILLO-PBSS was already used in similar projects where targets were successfully identified thanks to its ability to take into account protein flexibility^12^.

Among the Top-10 off-target proteins (out of 26,141) SPILLO-PBSS has recognized tubulin as a BTZ off-target. Conversely, tubulin was not identified (835th ranking position) as an off-target for CFZ. We hypothesized a possible interference of BTZ with the balance that regulates MT dynamics that may account for the different neurotoxic profile between BTZ and CFZ.

As a part of cytoskeleton, MT are dynamic and structural cellular components responsible for several cell functions. In neurons, the MT of axons and dendrites provide a structural backbone to maintain their proper morphology, and they are essential for the neuronal migration, polarity and differentiation^25^. In addition, neuronal MTs are essential for cargo trafficking, which is driven by motor proteins that can move vesicles and organelles along MT^8^.

Reflecting the importance of MT cytoskeleton in neuronal development, the involvement of direct targeting of MT by microtubule-targeting agents (MTA), which results in neurotoxic side effects, is now an emerging field of investigation.

For example, it is largely accepted that taxane, epothilone and vinca alkaloids, known drugs used to treat many paediatric and adult malignancies, commonly upset the microtubule dynamics leading to axonal degeneration^16^. Indeed, the impairment of the affinity between tubulin and motor protein or microtubule-associated protein (MAP) induced by MTA, could be one part of the mechanism that leads to neurotoxicity^26,27^.

Moreover, the impairment of axonal transport, which strongly depends on the MT system, is a common molecular target of the neurotoxicity of other classes of chemotherapy drugs, including BTZ^28^, cisplatin^29^ and oxaliplatin^30^. Our findings are consistent with reports of a relevant role of tubulin damage in the alteration of axonal integrity and development of CIPN^2,7,9^. Furthermore, a recent article shows that blocking the motor protein kinesin-5 by co-treatment with monastrol could resolve the neurotoxicity, by alleviating morphological measures of axonal injury in C57BL/6 mice treated with BTZ^31^.

Literature data support BTZ-induced perturbation of microtubule dynamics. In fact, BTZ-induced tubulin polymerization has already been demonstrated in multiple myeloma cells (HCN2 and RPMI 8226), renal carcinoma cells (786-0), neuroblastoma cells (KCNR and SH-SY5Y)^32^ as well as in E15 rat DRG neurons^7,28^, in adult rat DRG neurons and in the sciatic nerve of BTZ-treated rats^7^. Therefore, the identification of tubulin as off-target by SPILLO-PBSS and the experimental evidence of BTZ direct binding to tubulin by NMR, allow us to demonstrate for the first time that BTZ neurotoxicity is directly correlated to interaction between BTZ and microtubules.

Our results of tubulin polymerization in in vitro assay seem only apparently in contrast with data not shown by Porunchynsky et al. which make them declare that the BTZ effect on microtubules must be indirect^32^. In fact, also in our experimental cell-free model, BTZ does not induce polymerization but reduces the cold-induced depolymerization. This result highlights the different modes of action between BTZ and Paclitaxel. Actually, our hypothesis, supported by data obtained in the cell-free model, is that the drug prevents the release of GTP by shifting the balance towards the MT polymerized form and consequently reducing microtubule catastrophe. In literature, the role of GTP and GDP-tubulin in microtubules curvature, dynamics and in polarized vesicle transport has already been addressed^33–35^. To corroborate our in silico hypothesis we also demonstrated the direct binding of BTZ to tubulin by NMR ligand-receptor interaction studies. STD NMR is a very robust approach extensively used to detect and characterize molecular interactions between proteins, or their assemblies, and bioactive compounds^36–39^. Our STD experiments clearly revealed the binding of BTZ to tubulin in both its dimeric and polymeric form. On the contrary, CFZ did not show the same behaviour.

The value of the results is even more significant considering that CFZ is not able to increase the rate of tubulin polymerization and of GTP-tubulin both in DRG sensory neurons and in cell-free in vitro assay.

This multidisciplinary study asserts and gives evidence for an additional differential molecular mechanism of action of BTZ and CFZ that would explain their different neurotoxicity. Moreover, results obtained in DRG sensory neurons primary culture demonstrate that BTZ neurotoxicity is not related to its well-known antineoplastic effect but reasonably to its ability to directly bind to tubulin, reducing microtubule catastrophe and consequently increasing the rate of polymerized tubulin. Moreover, an increase in tubulin polymerization observed in multiple myeloma cells suggests that the interaction BTZ-tubulin could contribute also to BTZ antineoplastic effect. To further strengthen our hypothesis that the off-target interaction between BTZ and tubulin may play a role in the onset of BTZ induced PN, future experiments should be performed to demonstrate the effective BTZ-tubulin binding also in DRG neurons.

## Materials and methods

### Cell culture

U266.B1 human multiple myeloma cells (ATCC® TIB-196™) were cultured in RPMI 1640 medium supplemented with 10% fetal bovine serum, 1% L-glutamine, 1% Penicillin and Streptomycin (Euroclone, Italy). Cells were incubated at 37 °C and 5% CO_2_ in a humidified incubator.

### Adult mice dorsal root ganglia sensory neurons primary culture

Neuron cultures were obtained from C57BL/6 male mice of 8 weeks. All experimental procedures were carried out in compliance with the Animal Research: Reporting of In Vivo Experiments (ARRIVE) guidelines and in accordance with National Institute of Health guidelines for animal care and use of Laboratory animals (DL 2016, Italian Ministry of Health approval protocol 919/2015-PR). A complete dorsal root ganglia (DRG) pool was collected in F12 medium (Euroclone, Italy) from each mouse. DRG were digested for 1 h with 12.5 mg/ml collagenase (Sigma Aldrich, USA) and 10 mg/ml DNase (Sigma Aldrich, USA) and then mechanically triturated. A BSA gradient (30% BSA, 70% F12 medium) was used to isolate neurons that were then resuspended in BS medium (F12 medium supplemented with 1% N2 supplement 100X (Life Technologies, UK), 1% BSA (Sigma Aldrich, USA), 1% Penicillin and Streptomycin 100X (Euroclone, Italy), 1% L-glutamine 100X (Euroclone, Italy) and seeded in a single drop on poly-l-lysine (Sigma Aldrich, USA) coated dishes. After 24 h, neurons cultures were treated with 10^−5^ M 2′-Deoxy-5-fluorouridine (FuDR) (Sigma, USA) to remove satellite cells.

### Drugs

Bortezomib (BTZ, LC-Laboratories, USA), Carfilzomib (CFZ, AMGEN, USA) and Paclitaxel (PACLI, Cytoskeleton, USA) were solubilized in DMSO at a concentration of 2.6 mM, 5 mM and 2 mM respectively. Further working dilutions were made directly into culture medium.

### MTT assay

U266.B1 cells were seeded in 96-well plates at 10^4^ cells/well density. Cells were then treated with increasing concentrations of BTZ or CFZ (0.5–10 nM). After 48 h of treatment, a 5 mg/ml solution of 3-(4,5-dimethylthiazol-2-yl)-2,5-diphenyltetrazolium bromide (MTT) (Sigma-Aldrich, USA) was added directly to culture medium at a final concentration of 0.5 mg/ml. Plates were incubated at 37 °C for 4 h and centrifuged at 2 × 10^3^ rpm. Culture medium was removed and formazan crystals were solubilized in acidified 2-propanol (0.3% HCl). Absorbance of the solution was measured at 560 nm in a multiplate reader (BMG-Labtech, Germany).

### Trypan blue vital count

U266.B1 cells were seeded in 6-well plates at 250 × 10^3^ cells/well density. Cells were then treated with increasing concentrations of BTZ or CFZ (0.5–10 nM). After 48 h of treatment, cells were collected, stained with Trypan blue vital dye (Sigma-Aldrich, USA) and counted in a Burker hemocytometer. Both viable and dead cells were counted.

### Neurons viability and neurites measurement

Neurons survival was evaluated taking pictures of the same field, through a camera linked to an inverted microscope at day 0 and at 48 h. Viable neurons, characterized by a birefringent outline which is absent in dead cells, were manually counted using ImageJ software.The cell death induced by BTZ or CFZ treatment was calculated and compared to the ones obtained in the untreated control culture at different time points. Neurites elongation was measured in the same pictures used for neurons viability. At least 30 neurites for each picture were measured using NeuronJ plugin for ImageJ.

### Proteasome assay

Protein extracts to evaluate proteasome activity were obtained as described in protein extracts paragraph, but lysis buffer was made without proteases and phosphatases inhibitors (PMSF, Aprotinin, sodium pyrophosphate and sodium orthovanadate).

40 µg of proteins were loaded in black 96-well plates with 10 µL of 10X proteasome buffer (250 mM Hepes pH 7.5, 5 mM EDTA pH 8.0, 0.5% NP-40, 0.01% SDS) and 10 µl of proteasome substrate (7.6 mg/ml N-Succinyl-Leu-Leu-Val-Tyr-7-Amido-4-Methylcoumarin) (Sigma-Aldrich, USA). After 2 h at 37 °C, fluorescence was quantified in a microplate reader (Ex: 380 nm; Em: 460 nm) (BMG-Labtech, Germany).

### Protein database preparation

The protein database used for SPILLO-PBSS screenings (updated August 2019) included 26,141 entries retrieved from the RCSB Protein Data Bank (PDB)^10^, corresponding to protein 3D-structures experimentally solved by either X-ray diffraction or solution NMR, excluding 100% sequence identity redundancies. The collected entries included all available holo- and apo-proteins of the organisms *Homo sapiens* (20,976 entries), *Mus musculus* (3900 entries), and *Rattus norvegicus* (1265 entries), occasionally bound to protein chains of other organisms.

Biological assemblies for proteins showing multimeric structures were generated by the MakeMultimer program^40^, according to the BIOMT transformation matrices included into the PDB files. For multi-model PDB files from solution NMR experiments, only the first model was included in the database. No further protein structure refinements were needed to improve the quality of protein structures in the database.

### RBSs generation

The BTZ and CFZ reference binding sites (RBSs) used by SPILLO-PBSS to search the protein database for potential off-targets of the two drugs included 16 and 34 amino acid residues, respectively. They were obtained by molecular modelling techniques and the standard RBS generation protocol described in SPILLO-PBSS paper^11^. The amino acid composition of the two RBSs is reported in Supplementary Table S1.

### In silico screenings of the protein database

Two unbiased and systematic searches of the protein database for BTZ and CFZ potential off-targets were carried out by SPILLO-PBSS. Calculations were performed using a rotation step of 30° and a grid spacing of 2.0 Å, with the geometric tolerance set to 4.0 Å. Two distinct rankings were eventually obtained, one for each drug, in which the proteins are ranked according to their score, representing the similarity between the PBSs and the corresponding RBS. Proteins with the highest scores represent the potential off-targets of the drugs, as identified by the program.

### Protein extracts

#### Total protein extracts

U266.B1 cells were seeded and treated as described in the previous paragraph. After 48 h of incubation, cells were collected, washed with PBS, resuspended in 100 µl of lysis buffer (5 mM Hepes pH7.5, 150 mM NaCl, 10% Glycerol, 1% Triton X-100, 1.5 mM MgCl2, 5 mM EGTA, 4 nM PMSF, 1% Aprotinin, 20 nM Sodium pyrophosphate and 92 mg/ml Sodium orthovanadate) and vortexed for 30 s.

Neuron cultures were washed with PBS and 50 µl of lysis buffer was added to each dish. After mechanical scraping the suspension was collected.

Cell or neuron lysate was clarified with a centrifuge at the equivalent value 17,500 g for 15 min at 4 °C. Protein content was quantified using Bradford assay.

#### Fractionated protein extracts

Cells or neurons were lysed with lysis buffer (25 mM Tris HCl pH7.5, 1% Triton X-100, 5 mM EDTA pH8.0, 1 mM EGTA pH8.0, 10% glycerol, 4 nM PMSF, 1% Aprotinin, 20 nM Sodium pyrophosphate and 2 mg/ml Sodium orthovanadate). After chemical and mechanical lysis, the solution was sonicated and then centrifuged at the equivalent value 17,500 g for 15 min at 4 °C. The supernatant, which contains the soluble fraction of tubulin, was transferred to a new tube, while the pellet, which contains the polymerized fraction of tubulin, was resuspended and sonicated in lysis buffer supplemented with 0.5% sodium deoxycholate.

### Immunoblotting

Protein extracts were obtained as described in protein extracts paragraph with a complete lysis buffer.

10 µg of proteins were mixed with Laemmli buffer and then denatured at 95 °C for 5 min. Proteins were separated in a 13% acrylamide SDS-PAGE and, after electrophoresis, transferred to nitrocellulose filters for western blot analysis.

Membranes blocking, washing and antibody incubation were performed according to manufacturer’s instructions. Antibodies against β-tubulin (1:2000, Sigma-Aldrich, USA) and GTP-tubulin (1:1000, Adipogen, USA) were used. After incubation with primary antibodies, membranes were washed and then incubated with appropriate horseradish peroxidase-conjugated secondary antibodies (1:2000) (anti-mouse, Chemicon, USA; anti-rabbit, PerkinElmer, USA; anti-human, Sigma-Aldrich, USA). Immunoreactive proteins were visualized using an ECL chemiluminescence system (Amersham, USA).

### Cell-free tubulin polymerization and phosphate quantification

Cell-free tubulin polymerization in vitro was performed following manufacturer instruction (tubulin polymerization assay kit, Cytoskeleton). Briefly, the reaction solution was composed by the reaction buffer, 8% DMSO, 1 mM GTP and 2 mg/ml tubulin protein. The solution was mixed with drugs in 96-well plates and fluorescence was measured using a multiplate reader at different time points and at 37 °C (Ex: 340 nm; Em: 460 nm) (BMG Labtech, Germany).

Immediately after the end of the previous reaction, 50 µl of solution was transferred to a new 96-well plate and was mixed with 100 µl of Malachite Green reagent (Sigma Aldrich) to quantify the amount of free phosphate. Plate was then incubated at room temperature for 30 min and absorbance was measured at 620 nm in a microplate reader (BMG Labtech, Germany).

Tubulin depolymerization was evaluated after polymerization, placing the plate at 4 °C and measuring fluorescence every 5 min.

Four parameters were calculated on the results obtained in polymerization and depolymerization assays:

- Polymerization rate: defined as the increase in RFU% in one minute, in the exponential phase of the polymerization curve when placed at 37 °C.
- Polymerization lag: defined as the time required for the reaction to reach the exponential phase.
- Depolymerization rate: defined as the reduction in RFU% in one minute, in the exponential phase of the depolymerization curve.
- Depolymerization lag: defined as the time required for the reaction to reach the exponential phase when placed at 4 °C.

### NMR binding studies

NMR spectra were acquired on a Bruker AVANCE III 600 MHz NMR spectrometer equipped with a QCI (^1^H, ^13^C, ^15^ N/^31^P and ^2^H lock) cryogenic probe.

Samples for STD NMR experiments on tubulin assemblies were prepared by dissolving tubulin α/β dimers in a 20 mM PB buffer, MgCl_2_ 1 mM, pH 7.4, at a concentration of 10 μM or 20 μM. GTP was added at 100 μM concentration. BTZ or CFZ were dissolved in d_6_-DMSO and added to tubulin samples at a final concentration of 500 μM. d_6_-DMSO final concentration was 8% v/v. Each mixture was incubated at 37 °C for at least 30 min before NMR experiment acquisition. Samples for STD NMR experiments on tubulin dimers were prepared by dissolving tubulin α/β dimers in PB buffer, MgCl_2_ 1 mM, pH 7.4, at 10 μM concentration. BTZ, dissolved in D_2_O, was added to tubulin samples to a final concentration of 500 μM. To prevent tubulin aggregation, d_6_-DMSO addition was avoided and the mixture was kept at 10 °C before BTZ addition and during NMR spectra acquisition. Samples containing 500 μM BTZ or CFZ in 20 mM PB, pH 7.4, were also prepared to record ^1^H-NMR and STD NMR blank experiments to verify true ligand binding. Total sample volumes were 560 µL. The pH of each sample was measured with a microelectrode (Mettler Toledo, Columbus, OH, USA) and adjusted to pH 7.4 with small amounts (few microliters) of NaOD and/or DCl. ^1^H-NMR spectra were recorded (*zgesgp* pulse sequences in Bruker library) with 64 scans, spectral width of 14 ppm, relaxation delay of 3 s. STD-NMR spectra were recorded (*stddfiffesgp.3* pulse sequences in Bruker library) with 512 scans, spectral width of 12 ppm, saturation time of 2 s, on-resonance frequency = − 1.0 ppm or − 2.0 ppm, off-resonance frequency = 40 ppm. For STD, a train of Gaussian-shaped pulses of 50 ms each was employed to saturate selectively the protein envelope; the total saturation time of the protein envelope was adjusted by the number of shaped pulses. The on- and off-resonance spectra were acquired in an interleaved mode with the same number of scans. Each STD NMR spectrum was obtained by subtraction of the on-resonance spectrum from the off-resonance spectrum. All the spectra were processed with a line broadening of 0.8 Hz and corrected for phase and baseline.

### Statistical analysis

Each experiment was repeated at least three times and data are reported as the average ± standard deviation (SD). Statistical analysis was performed using GraphPad Prism 3 software. Differences between groups were evaluated using One Way ANOVA analysis of variance followed by Turkey’s multiple comparison test. Statistical significance was set at *p* < 0.05 and 0.01.

## Supplementary Information

Supplementary Information.
