# Supplementary material for: Tubulin binding potentially clears up Bortezomib and Carfilzomib differential neurotoxic effect
Source: Sci Rep. 2021 May 18;11:10523. doi: 10.1038/s41598-021-89856-3 (PMC8131610; doi:10.1038/s41598-021-89856-3)
Supplement: Supplementary file 1 — Supplementary Information. [file 41598_2021_89856_MOESM1_ESM.pdf]

Tubulin binding potentially clears up Bortezomib and Carfilzomib differential neurotoxic effect

Malacrida A., Semperboni S., Di Domizio A., Palmioli A., Broggi L., Airoldi C., Meregalli C., Cavaletti G., Nicolini G.

Supplementary information

Supplementary Table S1. Amino acid residues included in bortezomib (column A) and carfilzomib (column B) reference binding sites (RBSs).

(A)			(B)			
**Amino acid composition of bortezomib RBS**			**Amino acid composition of carfilzomib RBS**			
1st	THR A 1		1st	THR A 1	18th	ARG A 18
2nd	LYS A 2		2nd	THR A 2	19th	VAL A 19
3rd	LYS A 3		3rd	ASP A 3	20th	SER A 20
4th	GLY A 4		4th	GLY A 4	21st	TYR A 21
5th	ALA A 5		5th	TYR A 5	22nd	PRO A 22
6th	THR A 6		6th	SER A 6	23rd	SER A 23
7th	SER A 7		7th	ALA A 7	24th	ALA A 24
8th	GLN A 8		8th	ASP A 8	25th	ALA A 25
9th	ALA A 9		9th	ALA A 9	26th	TYR A 26
10th	PHE A 10		10th	GLY A 10	27th	SER A 27
11th	THR A 11		11th	LEU A 11	28th	ALA A 28
12th	ALA A 12		12th	ALA A 12	29th	TYR A 29
13th	ALA A 13		13th	PRO A 13	30th	VAL A 30
14th	GLY A 14		14th	GLY A 14	31st	ALA A 31
15th	GLY A 15		15th	ILE A 15	32nd	CYS A 32
16th	ALA A 16		16th	MET A 16	33rd	TYR A 33
/	/		17th	VAL A 17	34th	SER A 34

Supplementary Table S2. Percent sequence identities between *Ovis aries* tubulin alpha and beta chains and the corresponding tubulin alpha and beta isoforms of *Homo sapiens*, *Mus musculus* and *Sus scrofa*, as obtained by the basic local alignment search tool (BLAST, www.uniprot.org/blast/).

Tubulin isoform (UniProtKB AC)			***Ovis aries (Sheep)***	
			Alpha (D0VWZ0)	Beta (D0VWY9)
			Sequence identity %	
Homo sapiens	Alpha	1A (Q71U36)	100.0	/
		1B (P68363)	99.6	/
		1C (Q9BQE3)	98.0	/
		3C (P0DPH7)	97.6	/
		3D (P0DPH8)	97.6	/
		4A (P68366)	99.3	/
		3E (Q6PEY2)	96.0	/
		8 (Q9NY65)	89.8	/
	Beta	2A (Q13885)	/	99.6
		2B (Q9BVA1)	/	99.6
		3 (Q13509)	/	92.2
		4A (P04350)	/	95.5
		4B (P68371)	/	96.9
		6 (Q9BUF5)	/	91.4
		8 (Q3ZCM7)	/	88.3
Mus musculus	Alpha	1A (P68369)	100.0	/
		1B (P05213)	99.6	/
		1C (P68373)	98.2	/
		3 (P05214)	97.6	/
		4A (P68368)	96.0	/
		8 (Q9JJZ2)	90.0	/
	Beta	2A (Q7TMM9)	/	99.6
		2B (Q9CWF2)	/	99.6
		3 (Q9ERD7)	/	91.9
		4A (Q9D6F9)	/	95.9
		4B (P68372)	/	96.9
		5 (P99024)	/	95.7
		6 (Q922F4)	/	91.0
Sus scrofa	Alfa	1A (P02550)	99.3	/
		1B (Q2XVP4)	99.6	/
	Beta	(P02554)	/	98.7

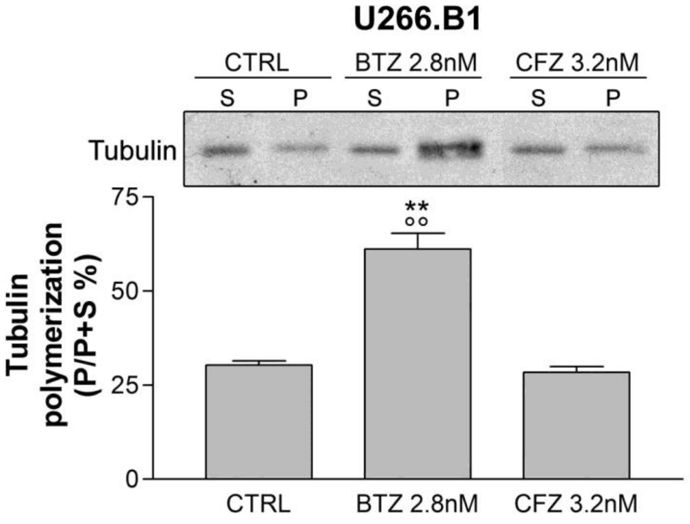

Supplementary Figure S1. Tubulin polymerization in U266.B1 cells. Representative images and quantification graphs of anti-tubulin immunoblotting in U266.B1 cells, not treated (CTRL) or treated for 48 hours with 2.8 nM BTZ and 3.2 nM CFZ. Graphs represent the percentage of polymerized tubulin (present in the pellet fraction P) compared to total tubulin (free tubulin, present in the substrate fraction S, added to polymerized tubulin). ** p<0.01 vs CTRL; °° p<0.01 vs CFZ 3.2 nM.

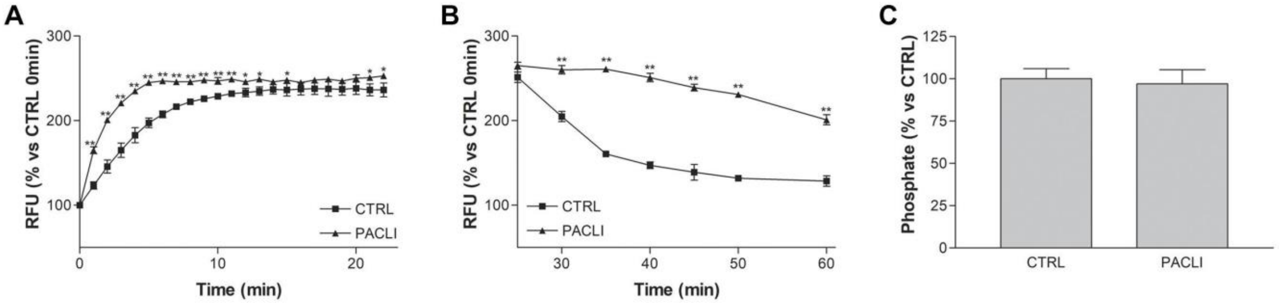

Supplementary Figure S2. Tubulin polymerization and depolymerization *in vitro* with DMSO buffer and PACLI. (A) *In vitro* cell-free tubulin polymerization in presence of 3 µM PACLI. (B) *In vitro* cell-free tubulin depolymerization in presence of 3 µM PACLI. (C) Phosphate quantification after tubulin polymerization assay. Graphs are represented as mean percentage ± SD compared to untreated CTRL (arbitrarily set to 100%). * p<0.05; ** p<0.01 vs CTRL.

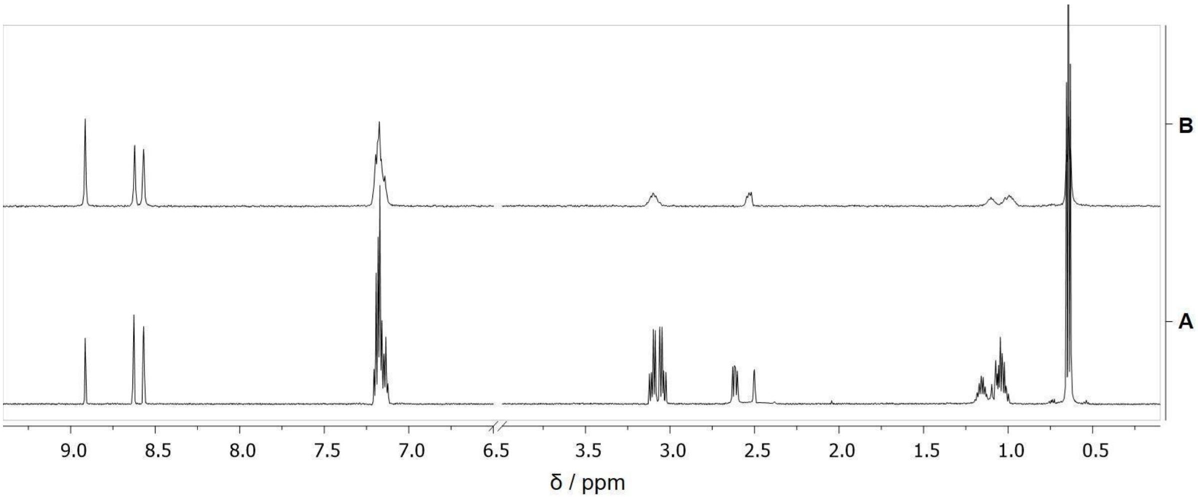

Supplementary Figure S3. A) ^{1}H NMR spectrum of 500 µM BTZ dissolved in 20 mM PB, $MgCl_2$, pH 7.4, 10 °C; B) STD NMR spectrum of 500 µM BTZ and tubulin α/β dimers 10 µM dissolved in 20 mM PB, $MgCl_2$, pH 7.4, 10 °C. All spectra were acquired at 600 MHz.

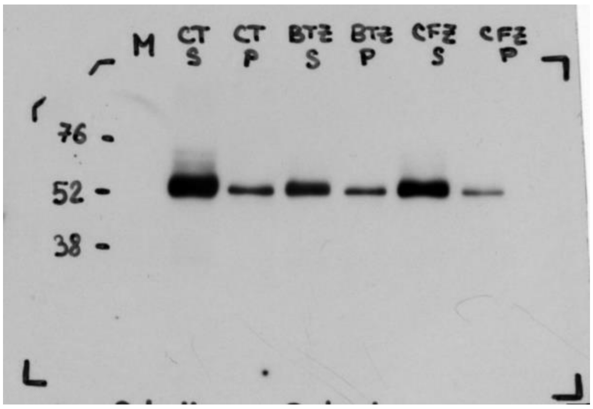

Supplementary Figure S4. Original immunoblotting image of Figure 6. Pellet/Supernatant tubulin immunoblotting.

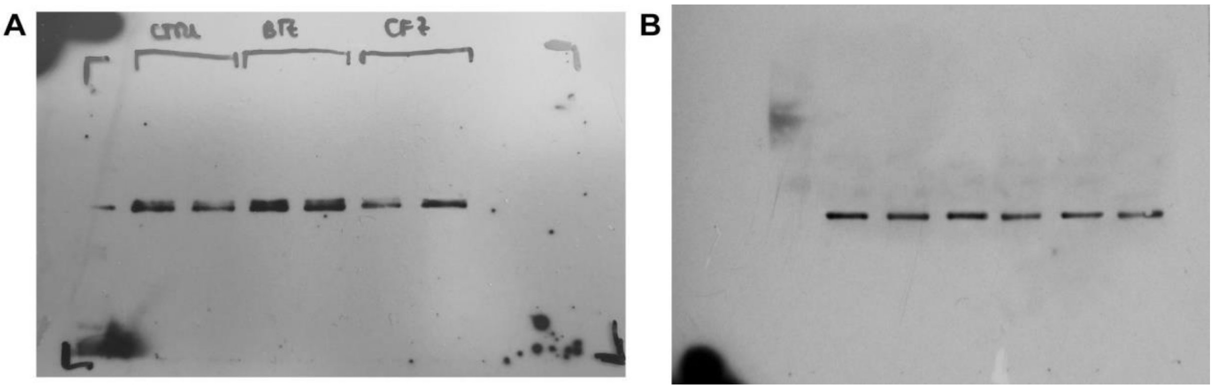

Supplementary Figure S5. Original immunoblotting images of Figure 7. GTP-Tubulin immunoblotting (A) and respective Actin immunoblotting (B).

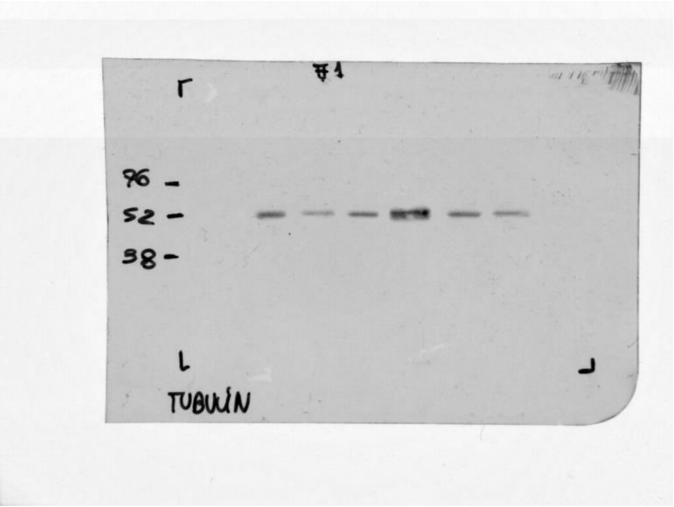

Supplementary Figure S6. Original immunoblotting image of Figure S1. Pellet/Supernatant tubulin immunoblotting.
